# Wandering spleen combined with pedicle torsion and splenic infarction: a rare case report and literature review

**DOI:** 10.3389/fped.2024.1429490

**Published:** 2024-09-16

**Authors:** Qian Cui, Xiaoyan Xu, Chang Li, Lihao Tang

**Affiliations:** ^1^ Department of Radiology, Shaoxing Central Hospital, Shaoxing, China; ^2^ Imaging Center, Tengzhou Central People’s Hospital, Tengzhou, China; ^3^ Department of Pathology, Tengzhou Central People’s Hospital, Tengzhou, China

**Keywords:** wandering spleen, torsion, infarction, contrast-enhanced computed tomography, acute

## Abstract

Wandering spleen (WS) concurrent with splenic pedicle torsion and infarction has been described rarely. We reported our experience in diagnosing and treating such a condition in a 16-year-old girl with acute abdominal pain. A plain CT scan showed the wandering of the spleen from the left upper quadrant. Contrast-enhanced CT indicated dilatation and distortion in the splenic vein, a counterclockwise “whirl sign” in the splenic pedicle, pancreatic tail torsion, and splenic infarction. The patient was diagnosed with WS combined with splenic pedicle torsion and splenic infarction and underwent splenectomy for treatment. She showed a satisfactory outcome during the follow-up. To enhance our understanding of it, we performed a comprehensive literature research to summarize the clinical manifestations, treatment options, and outcomes among adolescent patients.

## 1 Introduction

Wandering spleen (WS), also known as ectopic spleen, is a rare clinical condition in which the spleen migrates to an abnormal position within the abdomen or pelvis (1). It mainly affects children aged between 3 months and 10 years old and women of childbearing age (2). A major complication of WS is the torsion of the splenic pedicle, which can subsequently cause splenic infarction and rupture (3). In clinical practice, WS combined with acute splenic torsion is considered a life-threatening emergency (4). However, the diagnosis of this condition remains challenging due to its asymptomatic progression.

Currently, the diagnosis of WS mainly depends on clinical and imaging features, especially contrast-enhanced CT. This imaging technique can reveal the location, size, vascular condition of the spleen, and presence of splenic pedicle torsion. To date, less attention has been paid to the WS and the splenic torsion due to the rarity of cases. In this study, we present a case of a 16-year-old girl who presented with acute abdominal pain and was eventually diagnosed with WS, pedicle torsion, and splenic infarction.

## 2 Case presentation

**Figure 1:**
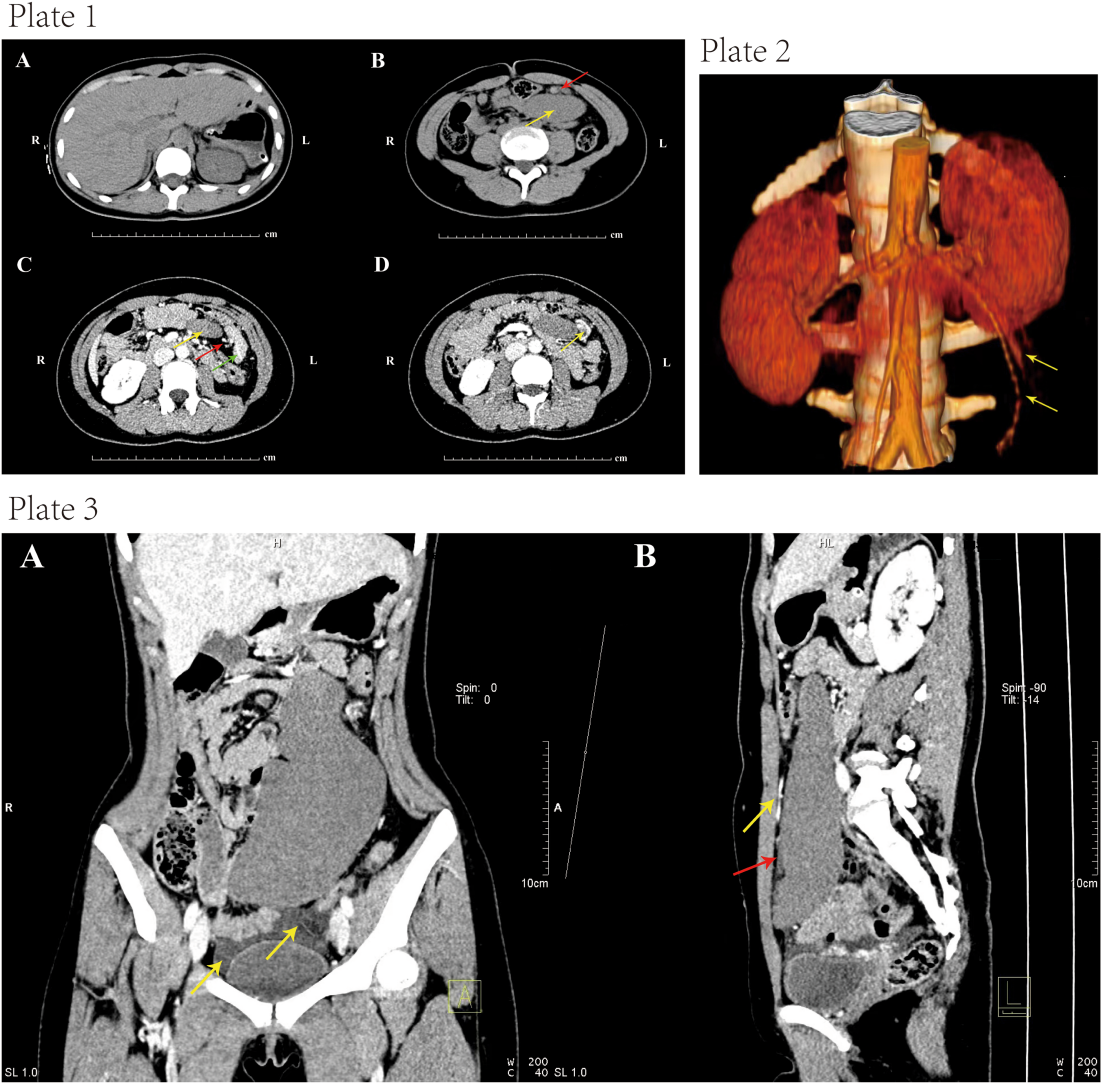
Plate 1, contrast-enhanced computed tomography (CECT) images. **(A)** Absence of the spleen in the left upper abdomen after plain CT scan. **(B)** CT scan indicated an elliptic soft tissue density shadow (yellow arrow) in the left upper quadrant. The hyperdense shadows in splenic veins suggested splenic vein thrombosis (red arrow). **(C)** The axial section showed a “whirl sign” (red arrow) of the splenic pedicle vessels counterclockwise, together with pancreatic tail torsion (green arrow). The white arrow indicated the absence of obvious enhancement in the spleen, showing a possibility of splenic infarction. **(D)** The visualized splenic veins showed dilatation and tortuosity (yellow arrow). Plate 2, computed tomography (CT) image showing elongated splenic pedicle vessels (yellow arrow). Plate 3, coronal and sagittal CT images. **(A)** Contrast-enhanced CT scan indicated splenomegaly. There was pelvic fluid induced by splenic infarction. **(B)** There was a filling of contrast agent in the upper segment of the vessels (yellow arrow), while there was no filling in the lower segment (red arrow). This indicated a block of the splenic artery.

A 16-year-old girl presented to our hospital’s emergency department with progressive aggravation of abdominal pain for at least 24 h. Physical examination revealed a migrating mass with tenderness in the left lower quadrant. A plain CT scan showed the spleen absent from the left upper quadrant (Figure 1, Plate 1A). In other areas of the left abdomen, a soft tissue density shadow in an elliptic shape was observed. The visualization of hyperdense splenic vessels suggested thrombosis of the splenic vein (Figure 1, Plate 1B). A contrast-enhanced CT scan showed a counterclockwise “whirl sign” in the splenic pedicle, along with pancreatic tail torsion and splenic infarction (Figure 1, Plate 1C). Furthermore, the visualized splenic veins showed dilatation and distortion (Figure 1, Plate 1D). Vascular reconstruction showed an elongated splenic pedicle vessel (Figure 1, Plate 2). The CT scan revealed an enlarged spleen with no enhancement in the arterial and venous phase, suggesting splenic ischemia and infarction (Figure 1, Plate 3).

The patient was finally diagnosed with WS combined with splenic pedicle torsion and splenic infarction. Laparoscopy was then performed in a supine position after anesthesia. A 10 mm trocar was inserted through an arc-shaped incision below the umbilicus. Carbon dioxide was injected to establish pneumoperitoneum, with pressure set to 14 mmHg. The laparoscope showed a black and infarcted spleen without ligamentous fixation that was merely suspended by the vascular pedicles in the left lower abdomen. In addition, the splenic pedicle was twisted 720° counterclockwise, along with pancreatic tail torsion.

**Figure 2:**
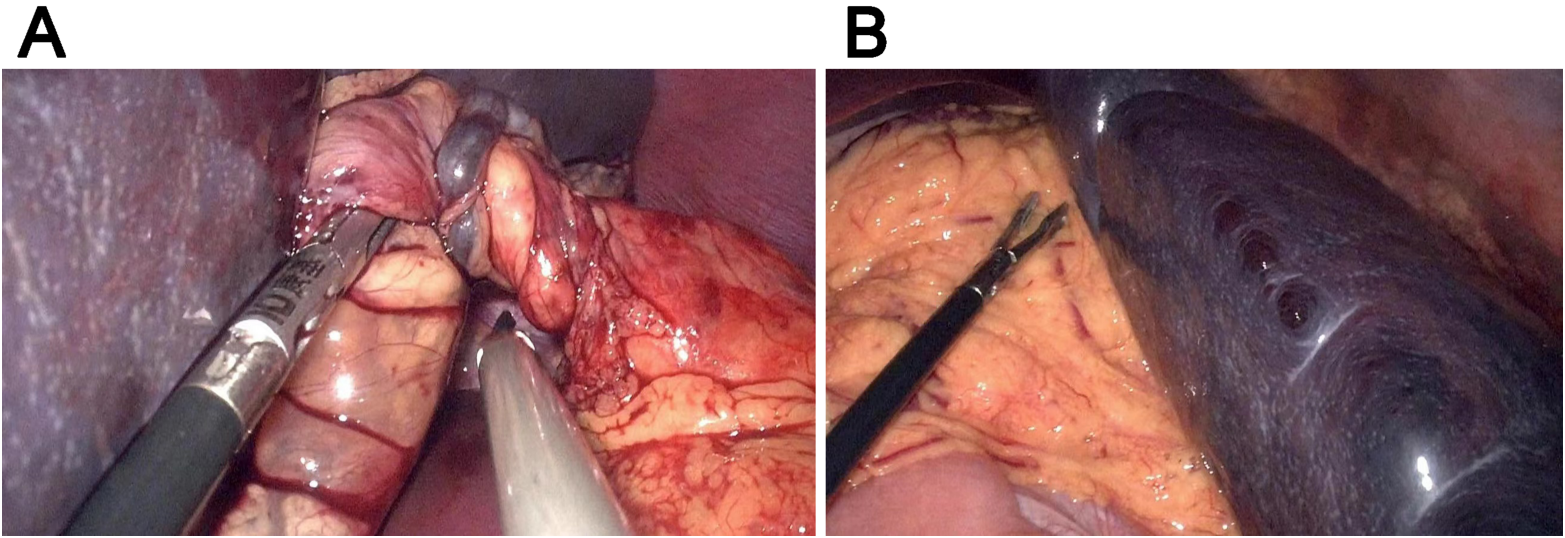
Representative intraoperative images. **(A)** The twisted splenic pedicle. **(B)** The splenic tissues of the spleen.

For the treatment, splenectomy (Figure 2) was performed since the splenic ischemia showed no improvement even after splenic repositioning. Three trocars (i.e., 10 mm, 5 mm, and 5 mm) were placed in the right lower abdomen, right umbilicus, and left upper abdomen, respectively. The spleen was repositioned to the splenic fossa, where it showed no adhesion to the surrounding structure and omentum. The splenic pedicle vessels were double-ligated at the pancreatic tail, and the splenic artery and vein branches were separated and cut using Hem-o-lock clips. The spleen was placed in a retrieval bag and was cut into pieces, and then the pieces were removed through the umbilical incision. The abdominal cavity was flushed, and the splenic blood vessels were ligated. A drainage tube was placed in the left upper abdomen and exited through the left trocar hole. The surgical time was 200 min, and the intraoperative bleeding volume was ∼30 ml. Postoperative histopathological analysis confirmed ischemic necrosis. During the 10-month follow-up, the patient showed normal conditions with no recurrence of abdominal pain.

## 3 Discussion

WS with torsion of the splenic pedicle is an extremely rare condition with an incidence rate of <0.2% (5). There is no sex difference under the age of 10 years; however, after the first decade, females have a higher prevalence of WS than that in males, with a ratio of 7:1 (6). Nevertheless, the diagnosis of WS combined with splenic pedicle torsion in adolescents remains challenging due to the rarity of cases. In this study, we report a 16-year-old girl with WS accompanied by splenic pedicle torsion and splenic infarction. Meanwhile, a comprehensive literature review was undertaken to fully enhance our understanding of this condition.

Anatomically, the spleen is fixed by six primary suspensory ligaments (i.e., gastrosplenic, splenorenal, splenophrenic, splenocolic, splenopancreatic, and presplenic fold ligaments) and two ligaments (pancreaticocolic and phrenicocolic ligaments) in an indirect association. The gastrosplenic, splenorenal, and phrenicocolic ligaments are usually loose or absent in the etiology of WS (2). Congenitally, WS is caused by the absence or deficits of ligaments resulting from the failure of the dorsal mesentery to fuse with the posterior abdominal wall during embryogenesis, which can be accompanied by developmental abnormalities such as congenital diaphragmatic hernia or intestinal malrotation (7). Additionally, the laxity of the supporting ligaments has been identified as an acquired cause of WS. This condition was mainly presented in women of childbearing age, secondary to multiple pregnancies and hormonal changes (8). Other acquired causes included malaria-, infectious mononucleosis- or lymphoma-induced chronic splenomegaly, abdominal trauma, and surgery (9).

Congenital WS is more common in adolescents than in adults. Cases are generally asymptomatic or show mild symptoms in childhood as the WS does not twist in the early years. The risk of WS increases with age and exercise. In adulthood, the prevalence of WS increases, with many patients developing acquired WS. In this study, we present a case of a 16-year-old girl with acute abdominal pain due to WS and splenic pedicle torsion. Therefore, we focused on the literature search on this condition in adolescents rather than adults. Herein, we identified 32 (10–41) previous case reports and case series published from 2003 to 2023 involving 37 WS cases (female, 25; male, 12) aged 12–18 years, as presented in Table 1. There was no difference in the sex ratio under the age of 10 years, but females showed a higher prevalence of WS than that in males (6).

**Table 1:** Thirty-seven adolescent patients with wandering spleen, from 2003 to 2023.

First author, year (Ref.)	Case No.	Gender/age (year)	Past medical history	Clinical and imaging findings	Diagnostic imaging approaches	Wandering position	Pedicle torsion	Treatment	Outcome
Alimoglu (10)	01	M/17	Hydrocephalus Ventriculoperitoneal shunt Chronic constipation Mental retardation	Abdominal pain and distention Low-grade fever Leukocytosis of 14 000/mm^3^ Massive colonic gas Intestinal adhesions Dilated small intestine Dolichocolon	Abdominal x-ray US	Right lower abdomen	Yes, 360°	Splenectomy Postoperative polyvalent pneumococcal vaccine and penicillin	Sigmoid colon resection was performed 3 months after splenectomy Uneventful recovery
Tucker (14)	02	F/17	4-year history of abdominal pain	Recurrent episodes of cramping pain in various regions of the abdomen Splenomegaly, hypersplenism with thrombocytopenia	CT	Mid-abdomen	Yes	Splenectomy	Uneventful recovery
Kleiner (12)	03	F/12	Mental retardation Myotonic dystrophy	Non-specific abdominal pain Normal size spleen Unremarkable palpation	US Radionuclide scan Laparoscopy CT	Left lower abdomen	Yes, clockwise 180°	Laparoscopic splenopexy	Uneventful recovery
Falchetti (11)	04	F/14	No	Mobile, slightly tender mass in the left lower abdomen Non-enhanced splenic cyst	US CT CECT	Lower pole of the left kidney	No	Laparoscopic splenopexy	Uneventful recovery
Upadhyaya (15)	05	F/16	No, but ride dirt bikes extensively	Symptoms of dysmenorrhea Large, mobile, well-vascularized mass 22 cm spleen extending from the level of the mid-pole of the left kidney to the pelvis, with a cyst measuring 4.6 × 2 cm in its inferior pole	US CT	Pelvis	No	Laparoscopic splenopexy Cystectomy	Uneventful recovery
Soleimani (13)	06	M/18	No	Intermittent abdominal pain + mass	US CT	Not available	No	Splenectomy	Uneventful recovery
	07	M/12	No	Tender abdominal mass	US	Not available	No	Splenectomy	Uneventful recovery
	08	F/14	No	Intermittent abdominal pain + mass	US	Not available	No	Splenectomy	Uneventful recovery
	09	F/12	No	Abdominal pain + mass	US CT	Not available	No	Splenopexy with absorbable mesh	Uneventful recovery
Feroci (25)	10	M/15	No	Diffuse abdominal tenderness and guarding Large hypogastric abdominal mass White cell count of 19.4 × 10^9^/L, hemoglobin 11.0 g/dl, and platelets 410 × 10^9^/L Splenic infarction and vein thrombosis	US CECT	Left lumbar and iliac region	Yes, 720°	Splenectomy	Uneventful recovery
Cho (21)	11	F/14	Vague recurrent periumbilical pain	Homogenous hypoechoic mobile mass Stomach distended into the left upper abdomen	US CT CECT	Left lower abdomen	No	Splenopexy	Uneventful recovery
Lien (31)	12	M/14	No	Acute abdominal pain, intensified by lying down, and relieved by standing up or walking around Depressed platelet count of 106 × 10^3^/μl Severe gastric spasm Splenic infarction and vein thrombosis	US Gastroendoscopy CECT Multi-detector row CT (MDCT) angiography	Anterior aspect of the middle to lower abdomen	Yes, 540°	Laparoscopic splenectomy Preoperative conjugate vaccines for pneumococcus and *H. influenza* type B Postoperative penicillin V	Uneventful recovery
Zarroug (38)	13	F/16	Prematurity (30 weeks)	Vague abdominal pain Malaise and vomiting for several weeks Mobile lower abdominal mass Mild pancytopenia	US CT	Pelvis	Yes, greater than 360°	Laparoscopic splenectomy	Uneventful recovery
	14	F/12	No	Non-tender abdomen with a 10 cm fixed mass in the infraumbilical region	US MRI	Pelvis	Yes	Splenectomy Postoperative aspirin for ∼2.5 months	Uneventful recovery
Yılmaz (37)	15	F/15	No	Sharp and persistent pain in abdomen Irritability and loss of appetite Enlarged spleen with partial infarction	US CT	Midline just below the umbilicus	Yes, counterclockwise 540°	Splenectomy	Uneventful recovery
Holst (27)	16	F/16	No	Acute right lower quadrant abdominal pain No appreciable mass and peritoneal signs	CT Laparoscopy	Right lower pelvis	Yes, 720°	Laparoscopic splenectomy	Uneventful recovery
Lombardi (32)	17	M/12	No	Tense abdomen with large palpable mass in pelvis Reduced hematocrit values Enlarged spleen with a large lesion arising from its inferior pole Separated bowel loops	Radiographs Doppler US CECT	Mesogastrium, left iliac fossa	Yes, 360°	Splenopexy	Not available
	18	M/15	No	Asymptomatic Large congested spleen with a sign of venous thrombosis	X-ray Color Doppler US CECT	From the medial margin of the left kidney to the pelvis	Yes	Splenectomy	Not available
Katsura (30)	19	F/13	No	Slightly tender, mobile mass in the left lower abdomen	CT	Left lower abdomen	Yes, counterclockwise 360°	Laparoscopic splenectomy Splenic autotransplantation Pneumococcal vaccination	Uneventful recovery
Rellum (34)	20	F/14	No	3 weeks of recurrent abdominal pain, malaise, and fatigue without fever Palpable spleen in the normal left upper abdomen	Color Doppler US MRI	Pelvis	Yes, clockwise 360°	Laparoscopic splenopexy	Uneventful recovery
Gorsi (26)	21	F/16	No	Severe abdominal pain Nausea and recurrent episodes of vomiting Distended fluid-filled stomach Splenic infarction, gastric volvulus (mesenteroaxial), and pancreatic volvulus	US CECT	Right side below the liver	Yes	Splenectomy Gastropexy Sham gastrojejunostomy	Uneventful recovery
Torri (36)	22	F/13	No	7 days of pain in the left abdomen and hypochondrium Fever and nausea Large palpable mesogastric mass, without tenderness and guarding A twofold increase of seric amylase and lipase count Splenomegaly and pelvic effusion Distal pancreatic torsion	US CECT	Mesogastrium	Yes	Laparoscopic splenopexy	Uneventful recovery
Jiang (29)	23	F/17	No	Intermittent lower abdominal pain Neutrophilia and leukocytosis Global splenic ischemia Cystic formations at hilar level	CECT	Pelvis	Yes	Splenectomy	Uneventful recovery
Aguirre Pascual (17)	24	M/18	No	Left flank bulge, nausea, and slight left lower quadrant pain Decreased platelet count Increased white blood cell count	Doppler US CEUS CT MRI	Left lower abdomen	Yes	Laparoscopic splenectomy	Uneventful recovery
Hui Lian (28)	25	M/16	No	Lower abdominal pain Palpable mass over the suprapubic region Mildly tender with dullness on percussion Leukocytosis	US	Pelvis	Yes, clockwise 720°	Splenectomy Triple vaccination against pneumococcus, meningococcus, and *Haemophilus influenza* 2 weeks after surgery	Uneventful recovery
Rizzuto (35)	26	F/17	Minor beta thalassemia	Leucopenia The triad of classic physical examination findings Coexistence of wandering spleen ad accessory spleen	US CECT	Right lumbar region to pelvis	No	Laparoscopic splenectomy Postoperative low molecular weight heparin (LMWH) for 30 days and triple vaccination against pneumococcus, meningococcus, and *Haemophilus influenza*	Uneventful recovery
Asafu Adjaye Frimpong (19)	27	F/14	Milder episodes of abdominal pains since childhood	5-day history of nausea, vomiting, loss of appetite, fever, headache, constipation, and central abdominal pain Tenderness around umbilicus Hypokalemia of 3.2 mmol/L and significant ketonuria Organoaxial gastric volvulus, with volvulus of the pancreas, wandering spleen, cholestasis Acute small bowel obstruction secondary to an ileo-ileal intussusception after the first laparotomy	CECT	Anteromedial of the abdomen	No	Laparotomy (twice)	Not available
Assaf (20)	28	F/13	No	Generalized, acute abdominal pain Fever, anorexia, and vomiting Spleen was infarcted without any ligamentous attachments	US CT	Hypogastric region	Yes, 540°	Splenectomy	Uneventful recovery
Abaszadeh (16)	29	M/15	No	Abdominal pain, right lower quadrant pain, pain under his bladder, pelvic pain, rebound tenderness, fever, nausea, vomiting, and lack of appetite Cystic lesion Appendix in the upper right quadrant	CT	Pelvis	Yes	Splenectomy Postoperative vaccination	Uneventful recovery
Dangen (24)	30	F/17	No	Firm abdominal mass Thrombocytopaenia Very large congenital diaphragmatic hernia Hypoplastic left lung and left pulmonary Left pneumothorax	US CT X-ray	Mid-abdomen	Yes	Splenectomy Postoperative vaccination and lifelong amoxicillin	Uneventful recovery
Perez-Rosillo (33)	31	F/18	Left diaphragmatic hernia repair two years earlier	High levels of C-reactive protein and leukocytosis Low-enhancing, comma-shaped mass in the hypogastric region	US CT	Hypogastric region	Yes	Splenectomy	Uneventful recovery
Colombo (23)	32	F/18	Wandering spleen	Frequent episodes of lower abdominal pain Multi-infarcted pelvic splenomegaly Normocromic normocytic anemia and piastrinopenia *Helicobacter pylori* gastritis Partially twisted pancreatic tail	CT Laparoscopy	Pelvis	Yes	Laparoscopic splenectomy Triple vaccination against *Haemophilus influenzae*, pneumococcus, and meningococcus 3 weeks before surgery	Uneventful recovery
Chue (22)	33	F/18	No	2-month history of intermittent left abdominal pain Palpable splenomegaly Gastric varices with features of portal hypertension	CT Oesophagogastroduodenoscopy	Pelvis	No	Laparoscopic splenectomy	Uneventful recovery
Ahmed (18)	34	M/12	Appetite loss, constipation, and mucoid diarrhea	Crampy lower abdominal pain Tachycardia (pulse rate of 134 beats per min) and respiratory rate of 22 breaths per minute Firm, tender mass across the left lower quadrant White blood cell count 10,300/micl with a neutrophilia of 78.5%, with moderate anemia (Hg 8.6 g/dl, Hct 28%), MCV 86 fl and platelet 775 × 103/micl Secondarily inflamed appendix	US	Lower abdomen	Yes, 720°	Splenectomy Appendectomy Vaccination against pneumococcus and meningococcus Antibiotic prophylaxis	Uneventful recovery
Bairwa (39)	35	F/14	No	Abdominal mass for ∼7 months Mild tachypneic Acute abdomen, abdominal distension, tenderness, rebound tenderness, diffuse guarding, and rigidity Increased platelet count Splenic ischemia and infarction	US CECT	Mid-abdomen	Yes, 720°	Splenectomy Triple vaccination against *Haemophilus influenzae*, pneumococcus, and meningococcus	Uneventful recovery
Petroianu (41)	36	M/14	Long-term history of frequent episodes of progressively worsening lower abdominal pain associated with dysuria, tenesmus, and constipation	Visible tender pelvic mass A reduced platelet count of 114 × 103/μl, a white blood cell count of 6.2 × 103/μl, and a hemoglobin level of 11.2 g/dl	CT CECT	Pelvis	Yes	Splenopexy	Uneventful recovery
Lugo-Fagundo, 2022 (40)	37	F/16	No	Severe periumbilical abdominal pain Wandering spleen with torsion and infarction	CT Laparoscopy	Right lower abdomen	Yes, 720°	Splenectomy	Uneventful recovery

CT, computed tomography; CECT, contrast-enhanced CT; US, ultrasound; CEUS, contrast-enhanced ultrasound; MRI, magnetic resonance imaging.

The diagnosis of WS is usually a challenge in clinical practice. Its symptoms vary according to the size and location of the spleen. For example, in our literature review, some cases were completely asymptomatic in the early stage (11). Along with disease progression, migration of the spleen predisposes to the torsion of the lengthy and loosening of the splenic pedicle. In the presence of mild torsion, the thin-walled splenic vein and its tributaries were first affected, resulting in congestive splenomegaly, abdominal discomfort or intermittent abdominal pain (13), and enlarged palpable mass with tenderness. Regional portal hypertension and gastric varices bleeding may occur in a long-term incomplete torsion (22). Moreover, the chronic splenomegaly can induce complete torsion. On this basis, the splenic artery blood flow will be blocked, and the spleen will be infarcted. The clinical symptoms usually include acute abdominal pain, hypotension, and even shock, accompanied by peritoneal irritation (31). The additional signs include fever, nausea, vomiting, loss of appetite, and a palpable mass in the mid-lower abdomen or pelvic cavity. Our case showed acute abdominal pain when presenting to our department. Indeed, attention should be paid to the differential diagnosis of WS from other diseases with acute abdominal pain as the initial symptom.

Laboratory tests for WS patients are mostly normal and non-specific, and occasionally, some patients may present with leukocytosis (10, 17, 28, 29, 33), thrombocytopenia (14, 17, 24, 31, 41), and neutrophilia (18, 29). Additionally, two patients (i.e., Patients 13 and 17) (32, 38) exhibited mild pancytopenia. One patient (i.e., Patient 22) (36) showed a twofold increase in serum amylase and lipase counts. Another patient (i.e., Patient 31) (33) showed an increased C-reactive protein level. Our case showed normal laboratory indicators, which implied that these laboratory indices may somehow contribute to the diagnosis of WS, but with low efficiency.

WS is usually confirmed by imaging methods such as plain x-ray (42), Doppler ultrasonography (USG) (43), CT (44), and magnetic resonance imaging (MRI) (39). In addition to these methods, one study (17) applied contrast-enhanced ultrasound (CEUS) for the diagnosis of WS. The images were characterized by the absence of the spleen in the left upper quadrant and the presence of a soft tissue mass elsewhere in the abdomen or pelvis. Nevertheless, CT is still the modality of choice for the final diagnosis of WS (45) as it shows multiple advantages such as high sensitivity (46) for the identification of splenic pedicle torsion. In our literature review, 30/37 (81%) adolescents were diagnosed with WS by CT. For the incomplete torsion, CT showed congestive enlargement of the spleen, together with dilated and twisted splenic veins. For complete torsion, splenic artery blood flow blockage was observed after the CT scan, and some or all non-enhancement areas appeared in the spleen. This indicated partial or total splenic infarction. Splenic pedicle torsion is characterized by splenomegaly and abnormal orientation of the splenic hilum, and a “whirl sign” can be used to predict parenchymal organ torsion (44). The hyperdense splenic pedicle is indicative of the thrombosed splenic vein (47). Capsule sign and peritoneal effusion can indirectly reflect splenic infarction. The VR reconstruction would visualize the position of the torsion and the elongated splenic pedicle. Our case presented with WS, combined with complete torsion of the splenic pedicle and splenic infarction. Contrast-enhanced CT showed the absence of mass enhancement in the left lower quadrant, and it was necessary to differentiate mesenteric cysts, lymphangiocysts, and adnexal tumors. Additionally, pancreatic volvulus was also reported in Patients 21 (26), 22 (36), 27 (19), and 32 (23). There may be rare symptoms such as pancreatic tail necrosis and pancreatitis under the simultaneous torsion of the WS and the pancreatic tail. Our case showed torsion of the pancreatic tail. The splenic artery was a terminal artery without communication and was also the terminal arterial circulation. Therefore, the incidence of splenic infarction was higher than that of other organs. Theoretically, any disease that can form an embolism can lead to splenic infarction. In addition to splenic pedicle torsion caused by WS, the other common causes of splenic infarction in children included infection, blood system diseases, and autoimmune diseases, such as infective endocarditis, malaria, sickle cell anemia, infectious mononucleosis, chronic myeloid leukemia, lymphoma, antiphospholipid syndrome, and arteritis (48). In the future, attention should be paid to the exclusion of *in situ* splenic infarction before considering the possibility of WS with splenic pedicle torsion.

Splenopexy and splenectomy have been used for the treatment of WS. In our literature review, 27 (73%) patients underwent splenectomy. Among these patients, 22 (81%) cases presented with different degrees of splenic torsion (Table 2). Splenopexy is the preferred treatment option for WS when there is no splenic torsion or when there is normal circulation in the distorted splenic vessels or normal blood supply after repositioning the splenic torsion. It is worth noting that a long-term follow-up is necessary given the likelihood of postoperative recurrence of splenic torsion. Splenectomy is required for treating WS accompanied by complications such as splenomegaly, splenic infarction, rupture, and thrombosis (6, 49). However, there is a high possibility of septicemia and severe infection after splenectomy. In our literature review, Patient 1 (10) received polyvalent pneumococcal vaccine and penicillin after splenectomy. Patient 12 (31) received conjugate vaccines against pneumococcus and *Haemophilus influenza* type B before laparoscopic splenectomy, as well as postoperative penicillin V. Patient 14 (38) received aspirin for about 2.5 months after splenectomy. Three patients (i.e., Patients 25, 26, and 39) (28, 35, 39) received triple vaccination against pneumococcus, meningococcus, and *Haemophilus influenza* after splenectomy, while one patient (i.e., Patient 32) (23) received the same vaccine before splenectomy. Patient 30 (24) received lifelong amoxicillin. Therefore, it is recommended to use antibiotics and capsular bacterial vaccines (e.g., *Haemophilus influenzae*, meningococcal, and pneumococcal vaccines).

**Table 2:** Summary of surgical procedures for wandering spleen with and without torsion in the literature review.

Surgery	WS without torsion (*n* = 10)	WS with torsion (*n* = 27)	Total (*n* = 37)
Splenectomy	5 (50.0%)	22 (81.5%)	27 (73.0%)
Splenopexy	5 (50.0%)	5 (18.5%)	10 (27.0%)

## 4 Conclusion

We reported a rare case of WS combined with splenic pedicle torsion and infarction with acute abdominal pain as the initial symptom. WS with splenic pedicle torsion should be considered in the differential diagnosis in children who present with recurrent abdominal pain and a palpable abdominal mass. CECT is the first choice for early diagnosis of WS, which may improve the possibility of spleen preservation and prevent the occurrence of complications.

## Data Availability

The original contributions presented in the study are included in the article/Supplementary Material, further inquiries can be directed to the corresponding author.
